# Combining Animal Welfare With Experimental Rigor to Improve Reproducibility in Behavioral Neuroscience

**DOI:** 10.3389/fnbeh.2021.763428

**Published:** 2021-11-30

**Authors:** Cássio Morais Loss, Fernando Falkenburger Melleu, Karolina Domingues, Cilene Lino-de-Oliveira, Giordano Gubert Viola

**Affiliations:** ^1^Molecular and Behavioral Neuroscience Laboratory, Departamento de Farmacologia, Universidade Federal de São Paulo, São Paulo, Brazil; ^2^National Institute for Translational Medicine (INCT-TM), National Council for Scientific and Technological Development (CNPq/CAPES/FAPESP), Ribeirão Preto, Brazil; ^3^Departamento de Anatomia, Instituto de Ciências Biomédicas, Universidade de São Paulo, São Paulo, Brazil; ^4^Instituto de Investigação e Inovação em Saúde, Universidade do Porto, Porto, Portugal; ^5^Departamento de Ciências Fisiológicas do Centro de Ciências Biológicas, Universidade Federal de Santa Catarina, Florianópolis, Brazil; ^6^Independent Researcher, Mossoró, Brazil

**Keywords:** replicability, reduce, refine, laboratory animals, animal models, behavior, enriched environment, ethology

## Introduction

*Reproducibility* is an essential characteristic in any field of experimental sciences, this feature provides reliability to the experimentally obtained findings (for details, see Glossary). The currently available empirical estimates on the topic suggest that less than half (ranging from 49% down to 11%) of scientific results are reproducible (Prinz et al., 2011; Begley and Ellis, 2012; Freedman et al., 2015, 2017). While it can be argued that the accuracy of these estimations needs confirmation, we (as a scientific community) have to recognize that poor reproducibility is a major problem in the life sciences.

The perception of an undergoing “reproducibility crisis” has led to the establishment of crowdsourced initiatives around the world addressing reproducibility issues in sciences, such as behavioral neuroscience (Open Science, 2015; Freedman et al., 2017; Reproducibility Project and Cancer Biology, 2017; Amaral et al., 2019). Among the explanations for poor quality in published research, there is the prevalent culture of “reporting positive results” (publication bias) and the high incidence of diverse types of experimental bias, such as lack of transparency and poor description of methods, lack of predefined inclusion and exclusion criteria resulting in unlimited flexibility for deciding which experiments will be reported, insufficient knowledge of the scientific method and statistical tools when designing and analyzing experiments (Ioannidis, 2005; Cumming, 2008; Sena et al., 2010; Freedman et al., 2017; Vsevolozhskaya et al., 2017; Ramos-Hryb et al., 2018; Catillon, 2019; Neves and Amaral, 2020; Neves et al., 2020). Further discussions on the causes, consequences, and actions to overcome poor research practices and reproducibility in sciences are many (Altman, 1994; Macleod et al., 2014; Strech et al., 2020) and beyond the scope of this text. Here, we focus on the aspects relevant to the field of behavioral neuroscience, whereby poor research performance may affect not only the economic and translational aspects of science but also implies ethical issues once it involves necessarily living subjects, mostly laboratory animals (Prinz et al., 2011; Begley and Ellis, 2012; Festing, 2014; Freedman et al., 2015; Voelkl and Wurbel, 2021).

In our opinion, combining principles of animal welfare with experimental rigor may lead to improvement in the quality of studies in behavioral neuroscience. Hence, we will briefly discuss how adherence to legislations, guidelines, and ethical principles in animal research may guide more rigorous behavioral studies. Thereafter, we condense discussions on how (1) the better understanding of the conceptualization, validation, and limitations of the animal models; (2) the use of suitable statistical methods for study design and data analysis; and (3) the use of *environmental enrichment* in research facilities to favor welfare of animals may improve quality of studies in behavioral neuroscience (some practical tips in Table 1) and, hopefully, the reproducibility in the field.

**TABLE 1:** Practical tips combining animal welfare and experimental rigor to improve reproducibility in behavioral neuroscience*.

Category	What should we do	Examples, warnings and observations
*Before (study planning)*		
• Regulations	• Prepare a study protocol according to legal, ethical, and institutional regulations applied to the project. • Submit the protocol to the ethical committee before study onset.	• Usually, all procedures involving laboratory animals, from transportation to the arrival in the laboratory until the humanitarian endpoints, should be included in the protocol. • Consult your institution about regulations applied to your project. • Perform experiments and other procedures involving laboratory animals after institutional approval of the protocols to avoid ethical or legal issues.
• Experimental design	• Define the experimental groups, allocation ratio (if sampling will be balanced between groups or not), minimal biological effect size, statistical model, statistical power and alpha to calculate the sample size *a priori.* • Consider to use randomized block experimental designs is useful for controlling confounding factors-related variability • Define how allocation to the groups will be performed. • Define how blinding of experimenters will be performed. • Define inclusion and exclusion criteria beforehand, explain how sample size will be kept. • Register online the protocol and the experimental design you are going to execute	• PREPARE guidelines and electronic assistant EDA may help you to make a complete experimental and analytical plan. • *A priori* sample size calculation will help you to avoid *p-hacking* and *data dredging.* • Sequence generation and allocation concealment help you to avoid selection bias. • Randomized block designs are more powerful than completely randomized designs being in accordance with the “reduce” principle. • Blinding (or masking) of experimenter to animals‘ treatment and outcome assessments will help you to avoid performance and detection bias, respectively. • Predefined exclusion criteria help you to avoid *cherry picking* and attrition bias. • Make available (online) your planning will help you to avoid HARKing behavior (“Hypothesizing After the Results are Known”)
• Personnel training	• Training (any procedures with animals) under supervision. • Training on the routine procedures in the animal facility. • Training on the routine procedures in the experimental rooms.	• Novices should be instructed on how to dress in the animal facility or experimental rooms (use a dedicated lab coat; avoid circulating outside the animal facility with the dedicated lab coat; avoid use perfume or creams, shampoos, other products with fragrance; avoid lab coat washing with laundry softener or perfumed soap). • Novices should be instructed on how to behave in the animal facility or experimental rooms (avoid speak loudly or make sudden noises; avoid using headphones to listen to music or speak on • the mobile phone). • Activities planned to the experiment (transportation from a box/room/apparatus to another; injections, animal handling and restraining methods, blood samplings, surgeries; behavioral testing; etc.) should be rehearsed as much as necessary to be learned and make experimenters confident about all the steps trained. • Complete personal training before real experiments onset.
• Environment settings	• Check devices controlling animal room settings (light, temperature, humidity, light cycle) • Check availability of the resources to the home cage (bedding, enrichment, food/water availability) • Check devices controlling experimental room settings (light, temperature) • Advertise to all personnel and keep visible posters with the rules, procedures, and routines in the animal house and experimental rooms.	• Animal room settings, home cage conditions and experimental room settings should be decided according to species-specific homeostatic needs and experimental requirements as specified in the protocol approved by the ethical • committee. Restrict the access to the animal and experimental rooms to authorized, trained personnel. • Consider using environmental enrichment as the standard condition (see below), as recommended by the legislation.
• Animals and environmental enrichment	• Observe animals’ appearance (fur, color of skin, eyes, body weight) and specifications (species, strain, sex, age, number of animals, batch number) upon arrival in the laboratory. • Be aware of the characteristics of the species (and strain when applicable) that will be used in the study (rat, mouse, fish, worm, fly, etc.). • Keep animals in suitable (enriched) environments to improve animal welfare.	• Type (species, strain, sex, age) and number of animals should be as prespecified in the protocol approved by the ethical committee. • Perform periodical assessment and record of animals’ appearance (fur, color of skin, eyes, body weight, secretions, feces), behavior (general activity, food, and water intake) and home cage conditions (bedding, enrichment, food/water availability) during their stay in the animal facility. • Notify unexpected events to the staff responsible for the animal experimentation in the laboratory.
• Animal models and behavioral tests	• Choose an animal model suitable for testing the study’s hypothesis. • Be familiarized with the key features of your animal model, the particular behavioral and physiological characteristics and/or responses to interventions • Choose an appropriate behavioral assay to test your animal model or treatments/interventions administered to your model. Define the behavioral and physiological variables to be measured in your test.	• Understand the validity and limitations of the model in question. i.e., What aspects (if any) of a human pathology is modeled by your animal? Is it a model or a behavioral assay (e.g., tests design for drug screening). • Check the original source for the model or test development and validation process. Avoid citing or relying on subsequent work that may have misinterpreted the or modified the model or test parameters. • Does the test allows for the animal to express the expected behavioral/physiological output to be measured (e.g., in the case of models that present some kind of motor dysfunction, a test that relies heavily on motor function should be avoided).
*During experimentation*		
• Experimenters	• Prepare experimental settings. • Inform other personnel that behavioral experiments are in progress. • Make experimenter blind to animals’ treatment (or experimental group). • Follow the prespecified protocol.	• Use a dedicated lab coat (different of that used in the other sectors of the laboratory). • Use fragrance-free products in the body, hair, and clothes (including lab coat). • Record unexpected events and deviations of the protocol in the lab book (or equivalent)
• Experimental room	• Illumination, temperature, and level of noise in the experimental room should be set right before behavioral testing. • Position the video camera and video recording settings should be right before behavioral testing. • Organize an adjacent room with appropriated settings to receive experimental animals after behavioral testing.	• Time-to-time check the settings of experimental room, adjacent room and video recording devices during every experimental session (include this in your time schedule). • Only bring the experimental animal to the experimental room when the environment is prepared to the behavioral testing.
• Experimental procedures and data collection	• Identify animals according to the rule prespecified in the step of allocation to the groups. • Bring animals to the experimental room following the prespecified order (preferentially randomly selected) • Consider using automated methods (such as hardware and software) to collecting data.	• Blinding of animals’ treatments will help you to avoid performance bias. • Avoid bringing animals directly from the home cage to the experimental procedures, include an interval in the experimental room before behavioral testing. • Avoid returning animals directly from experimental procedures to the animal house or home cage where are untested animals. • Perform behavioral testing, or invasive procedures, in a laboratory animal away of the conspecifics (many species communicate using ultra-sonic vocalizations and scent). • Automatization of data collection helps to minimize observer bias and also between laboratory variations. It also mimics blinding procedure during data collection.
• Animals and environmental enrichment	• Observe animals’ appearance (fur, color of skin, eyes, body weight) and specifications (species, strain, sex, age, number of animals, batch number) throughout the experiment. • Keep animals in suitable (enriched) environments to improve animal welfare.	• Social animals must be kept in groups (except for procedures that require isolation). When keeping animals in groups, be careful not to compromise population density. • When using territorial animals (such as mice), avoid complete exchange of objects between home cage hygienization, as this can increase aggressiveness.
*After*		
• Animal models and behavioral tests	• When interpreting behavioral results from an animal model and/or behavioral assay, avoid overreaching conclusions and generalizations that extrapolate the model validity. What does the animal model behavioral response means? What does the test measured?	• For example, the administration of a certain drug, might reverse the immobility of mice subjected to chronic unpredictable stress (a model for depression) in the tail suspension test. This does not necessarily equals the reversal of depression. Since the measurement is immobility time, the drug might increase the overall activity of mice without having a true antidepressant effect.
• Analysis	• Follow the prespecified analytical plan. • Make blind assessment of the outcome when statistically analyzing the data. • Be careful about *outlier* exclusion • Make sure that the data met the assumptions for the chosen statistical method.	• Deviations of analytical plan and *post hoc* analysis should be acknowledged in the publication. • Blinding of outcome assessment will help you to avoid detection bias. • Do not include data obtained from animals that for some reason have experienced methodological problems (e.g., power outages during behavioral testing). They should not be treated (or tested) as *outlier* once deviation from normality is probably a consequence of problems during data acquisition. • When considering the exclusion of *outlier*, do it only after blinding the researcher to the groups. • Consider to use alternative statistical approach when the data does not met the assumptions for running the chosen statistical method. For example, using GLM as an alternative for ANOVA.
• Reporting	• Report hypothesis, methods and results transparently. • Make your data (including video recordings) available to the community. • Choose an adequate descriptive statistic to represent the data. • Report all the data collected (data points) • Report the effect sizes with confidence intervals	• ARRIVE guidelines may help you to make complete report of the study. • A complete report helps you to avoid reporting bias. • There are several data repositories where you can share your data with your peers. This is in accordance with the 3R principles once other researchers can use (explore) your data instead of performing a whole independent experiment again. • Consider to represent the data as median and range (instead of mean ± standard deviation) when data are not normally distributed. Alternatively, represent the data with confidence intervals (e.g., 95% CI). Avoid using standard error of the mean to represent data variability. • The confidence interval for the effect indicates how precisely the effect has been estimated • The effect size is a quantitative measure that estimates the magnitude of differences between groups, or strength of relationships between variables.

*Most of these practical tips may be found in manuals like Wolfensohn and Lloyd (2013), others are from authors’ own experiences with behavioral experiments. Other references used to prepare the practical tips: (Festing and Altman, 2002; Festing, 2014; Hooijmans et al., 2014; du Sert et al., 2017; Smith et al., 2018; Percie du Sert et al., 2020; Karp and Fry, 2021). Warning: In case of animal welfare concerns, experimenters and caregivers should always consult staff responsible for the animal experimentation in the laboratory (principal investigator, veterinary surgeon, lab manager).

## Advantages of the Adherence to the Regulations to the Quality of Behavioral Neuroscience

Behavioral studies in laboratory animals are performed worldwide under specific guidelines conciliating the needs of science, scientists, and animal welfare (Smith et al., 2018). Regulations establish obligations and responsibilities for institutional actors involved in animal experimentation, from students to deans (please consult one’s own institution about regulations applied to a project). Here, we claim that, besides being ethical, adherence to the regulations is advantageous to the quality of behavioral studies. Why? Because, regulations in animal research consider, among other things, the 3Rs principle (replace, reduce, and refine), which are the useful frameworks to prepare good quality experiments taking animal welfare into account, as discussed by previous authors (e.g., Franco and Olsson, 2014; Bayne et al., 2015; Aske and Waugh, 2017; Strech and Dirnagl, 2019) and in the further sections. “*Replace*” prompts scientists to consider alternatives to behavioral studies in laboratory animals for reaching a giving aim, in the first place. Once a behavioral study in laboratory animals is considered necessary, “*reduce*” may guide designs using well-established rules for rigorous experimentation to extract the maximum information of a study with a minimum number of subjects. The principle “*refine*” assists scientists to devise better strategies guaranteeing animal welfare according to species-sex-age-specific needs. There is evidence that “happy animals make better science” (Poole, 1997; Grimm, 2018). Besides, poor welfare in laboratory settings affects the laboratory animals in unpredictable, and often deleterious ways, compromising behavioral outcomes in the experiments (e.g., Emmer et al., 2018), and increasing the number of experimental animals unnecessarily. Therefore, personnel handling animals (experimenters, technicians, and caregivers) may contribute to the efforts to minimize the risk of animal suffering during procedures improving research quality. There are many free resources for training staff in the 3Rs principle made available by international organizations, such as NC3Rs^1^ or Animal Research Tomorrow,^2^ which could be easily implemented in behavioral studies.

## Suitable Animal Models and Behavioral Tests Should Improve Studies in Behavioral Neuroscience

The selection of an adequate animal model is a pivotal step in behavioral studies. *Physical models* (Godfrey-Smith, 2009) are central tools in neuroscientific research. Neuroscientists commonly employ *in vivo animal models*, aimed to simulate physiological, genetic, or anatomical features observed in humans (as is the case with studies of disease) or replicate natural situations under controlled laboratory conditions (van der Staay, 2006; Maximino and van der Staay, 2019). By definition, a model is a construct of a real physical component or property observed in nature. Therefore, a model is always imperfect and does not contemplate the full complexity of the real system that is being modeled (Garner et al., 2017). Much has been discussed about the validity and translational potential of animal models (Nestler and Hyman, 2010). Here, our aim is to consider how the misuse of animal models may affect the reproducibility and reliability of neurobiological research results. Firstly, there appears to be confusion about the definition of animal models and *behavioral tests* (Willner, 1986) that ultimately causes the misinterpretation of results. Animal models deliberately prompt changes in biological variables (such as behavior), while behavioral tests are paradigms in which animal models are subjected to having their behavior assessed. By this definition, a behavioral bioassay (an intact animal plus an apparatus) is not a model in a strict sense (van der Staay, 2006; Maximino and van der Staay, 2019), although useful to study normal animal behavior (e.g., exploration of a maze and immobility in forced swim test) and its underlying mechanisms (Maximino and van der Staay, 2019; de Kloet and Molendijk, 2021). Secondly, it is important to be aware of the conditions validated for the test because modifying some of them (e.g., light intensity or animal species/strain) may yield different results than those observed in the standardizations for the test (Griebel et al., 1993; Holmes et al., 2000; Garcia et al., 2005). For example, the dichotomic behavioral outcome (mobility or immobility) of mice is often registered in the tail suspension test. However, some mice (e.g., C57BL/6 strain) also present climbing behavior which may be mistaken by immobility (Mayorga and Lucki, 2001; Can et al., 2012). Third, we have to avoid the extrapolation of simple behavioral measures (those variables that we actually measure in a task) to complex multidimensional abstract behaviors (e.g., anxiety, memory, locomotor, and exploratory activities). For example, measuring only distance traveled (or the number of crossings) in an open field arena is not sufficient to fully capture the complexity of locomotor behavior (Paulus et al., 1999; Loss et al., 2014, 2015). Therefore, it alone does not provide enough information to make conclusions about locomotor activity, a multidimensional behavior that encompasses not only how much an animal moves (distance traveled and locomoting time) but also *how* it moves (average speed, number of stops made, among others) (Eilam et al., 2003; Loss et al., 2014, 2015). This extrapolation becomes even greater when we think about exploratory activity, which encompasses locomotor activity and other behaviors (such as time and frequency of rearing) (Loss et al., 2014, 2015). Similarly, Rubinstein et al. (1997) observed that mutant mice lacking D4 dopamine receptors moved less in the open field arena but outperformed their wild-type littermates in the rotarod test, which highlights that we cannot conclude much about motor function by measuring only the distance traveled (even if the amount of movement registered is similar between the groups). Finally, it is imperative to know whether the animal model we intend to test meets the assumptions of the behavioral paradigm (or our study hypothesis) that it will be tested. For example, animals with compromised mobility (e.g., models for spinal cord injury) will not provide meaningful results in tests that rely on preserved motor function (e.g., forced swim test, elevated plus maze). Similarly, subjecting a pigeon to the Morris water maze may lead one to conclude that pigeons have poor spatial memory. But, pigeons do not swim in the first place making the last experimental proposal not just inappropriate but absurd. Hence, knowledge about the biology of laboratory animals seems fundamental to the selection of a suitable approach for an intended behavioral study.

## Rigorous Design of Studies and Analysis of Data Should Improve the Quality of Behavioral Neuroscience

Limited knowledge of the scientific method and statistics are among the reasons for the high levels of experimental bias and irreproducibility (Ioannidis, 2005; Lazic, 2018; Lazic et al., 2018) leading ones to suggest that we are actually facing an “epistemological crisis” (Park, 2020). Several guidelines for experimental design, analysis, and reporting are available (see Festing and Altman, 2002; Lazic, 2016; Percie du Sert et al., 2020), describing rigorous methods that should be adopted to avoid bias achieving high-quality data production. However, it seems that some of the most basic good practices described in these guidelines have been neglected or ignored (Goodman, 2008; Festing, 2014; Hair et al., 2019). Some frequent sources of biases are *pseudoreplication* (Freeberg and Lucas, 2009; Lazic, 2010; Lazic et al., 2020; Eisner, 2021; Zimmerman et al., 2021) and violations of rules for experimental design, such as *a priori* calculating the sample size, unbiased allocation of samples to groups (randomization), blinded assessment of outcomes, complete reporting of results, and choosing the method for data analysis beforehand (Macleod et al., 2015). The lack of a rigorous plan results in the massive production of underpowered *exploratory studies* (Maxwell, 2004; Button et al., 2013; Lazic, 2018), with the aggravating factor that they are often misinterpreted as *confirmatory studies* ones (Wagenmakers et al., 2012; Nosek et al., 2018). It is not unusual to find discussions about the so-called “statistical trend” in studies in which both *biological effect sizes* and sample sizes are assumed *post hoc.* In addition, the extensive practice of exclusively using linear models (such as Student’s *t*-test or ANOVA) to analyze the data, assuming that all variables present Gaussian distribution, contribute to the misinterpretation of results (Lazic, 2015; Eisner, 2021). Currently, there are alternative methods that we strongly suggest to be incorporated in research projects by the whole neuroscientific community. For example, Generalized Linear (Mixed) Models and Generalizing Estimating Equations (GLM, GLMM, and GEE, respectively) fit distinct types of distribution (such as the Gaussian distribution) and correct for *confounding factors* (Shkedy et al., 2005a,b; Lazic and Essioux, 2013; Lazic, 2015, 2018; Bono et al., 2021; Eisner, 2021; Zimmerman et al., 2021). Adopting randomized block experimental designs (that are more powerful, have higher external validity, and are less subject to bias than the completely randomized designs typically used in behavioral research) is also necessary for controlling confounding factor-related variability and producing more reproducible results (Festing, 2014). Considering the use of multivariate statistical tools (instead of the widely used univariate approach) is an alternative to achieve more accurate outcomes from experiments with big data, especially in behavioral studies (Sanguansat, 2012; Loss et al., 2014, 2015; Quadros et al., 2016). Among the advantages of using these alternative approaches is the increased accuracy in parameter estimation (thus avoiding making *impossible predictions*), resulting in reduced probability of making Type I Error (due to invalid estimation of *p*-values, for example) and Type II Error (due to lack of statistical power). Rigorous design of studies and analysis of data should help to extract the maximum information of a study with the adequate calculated number of subjects and prevent waste of scientific efforts in behavioral neuroscience. In addition, rigorous and systematic reporting of methods (with enough details to allow replication) and results (with complete description of effect sizes and their confidence intervals rather than uninformative *p*-values) are also necessary to increase transparency and, consequently, the quality of the studies (Halsey et al., 2015; Halsey, 2019; Percie du Sert et al., 2020).

## Environmental Enrichment in Research Facilities May Favor Translational Neuroscience

As mentioned, “Happy animals make better science” (Poole, 1997; Grimm, 2018). It is a worldwide acknowledgment that environmental stimulus is necessary to improve the quality of life and welfare of captive animals, such as research animals. It has been more than a decade since the *Directive 2010/63/EU* was established (EC, 2010). However, this and other directives are far from being effectively complied with by the entire scientific community. A common non-tested argument to raise research animals in *impoverished standard conditions* is that the data variability among laboratories, or even within them, would increase by raising the animals in enriched non-standard conditions (Voelkl et al., 2020). This last claim has been criticized over the past two decades and suggested to be a fallacy (Wolfer et al., 2004; Kentner et al., 2021; Voelkl et al., 2021). For example, Wolfer et al. (2004) and Bailoo et al. (2018) observed that data variability did not increase after raising the animals in enriched environments when compared with raising them in standard laboratory environments. Furthermore, Richter et al. (2011) found that rearing animals in enriched environments decreased variation between experiments, strain-by-laboratory interaction on data variability. In other words, heterogenized housing designs appear to have improved data reproducibility. Therefore, it was claimed (and we agree) that we should embrace environmental variability (instead of static environmental standardization) because environmental heterogeneity better represents the wide variation (richness and complexity) of mental and physical stimulations in both human and non-human animals (Nithianantharajah and Hannan, 2006; Richter, 2017). In fact, drug development and discovery may be affected by the culture of raising animals in impoverished (extremely artificial) environments. There are studies showing that some drugs present biological effects when tested in animals raised in impoverished environments but not in animals raised in enriched environments (which is more similar to real-life conditions) (Akkerman et al., 2014; Possamai et al., 2015). Furthermore, we cannot disregard that more pronounced effects could be found whether drugs were tested in animals raised in enriched when compared to impoverished environments (Gurwitz, 2001). While one can argue that there are not enough studies strengthening this assertion, the low quality of life of captive animals, the low reproducibility of studies, and the poor translational rate of preclinical research reinforce the necessity of a *paradigm shift* related to the welfare of animals (Akkerman et al., 2014; Voelkl et al., 2020). This debate should not be restricted to rodents and shall include avians (Melleu et al., 2016; Campbell et al., 2018), reptiles (Burghardt et al., 1996), fishes (Turschwell and White, 2016; Fong et al., 2019; Masud et al., 2020), and even invertebrate animals (Ayub et al., 2011; Mallory et al., 2016; Bertapelle et al., 2017; Wang et al., 2018; Guisnet et al., 2021). We bring two practical examples (or recommendations) of improvements that we (the neuroscientific community) could do: (1) when using animal models we should implement environmental enrichment as the standard in the animal facilities (especially for those animal models that attempt to simulate central nervous system disorders), as raising animals in impoverished environments provides suboptimal sensory, cognitive and motor stimulation, making them too reactive to any kind of intervention (i.e., “noise amplifiers”) (Nithianantharajah and Hannan, 2006); (2) when proposing alternative organisms to study behavior (e.g., zebrafish), we should learn from past and present mistakes (mostly in rodents), keeping in mind the *ethological and natural needs of the species* (Branchi and Ricceri, 2004; Lee et al., 2019; Stevens et al., 2021). Importantly, when making these improvements we should carefully respect the species-specific characteristics. For example, rats and mice share some characteristics, such as nocturnal habits (which means that both species need places to hide during the light period, to provide a sense of security) (Loss et al., 2015). However, they also have some distinct characteristics, such as the need for running (which is higher in mice) (Meijer and Robbers, 2014). This means that providing running wheels for mice is really necessary, while for rats, (that run less but are more social than mice) (Kondrakiewicz et al., 2019) the space dedicated to some of the running wheels could be better used by increasing (carefully not to compromise the population density) the number of individuals in the home cage. On the other hand, zebrafish needs aquatic plants and several substrates in their environment, such as mud, gravel or sand, to represent their own eco-ethological expansions of behavior (Engeszer et al., 2007; Spence et al., 2008; Arunachalam et al., 2013; Parichy, 2015; Stevens et al., 2021). The substrates might provide some camouflage for zebrafish against the predator, which may contribute to feelings of security and improved welfare (Schroeder et al., 2014). Taking all these together, in our opinion, the scientific community must think over the long-term costs (economical and ethical ones) of keeping the culture of raising animals in impoverished environments, a condition that potentially disrupt the translation of behavioral neuroscience results into applicable benefits (Akkerman et al., 2014).

## Future Directions

As previously stated, a “reproducibility crisis” is not an issue limited to the field of behavioral neuroscience, and several crowdsourced initiatives were established around the world addressing reproducibility (Open Science, 2015; Freedman et al., 2017; Reproducibility Project and Cancer Biology, 2017; Amaral et al., 2019). An essential step to confront this issue is to first recognize that there is a crisis and that it is a major problem. Secondly, the scientific communities have been developing and disseminating guidelines for good experimental practices to be implemented by themselves (more information can be found in http://www.consort-statement.org/ and also in https://www.equator-network.org/). In addition, encouraging the preregistration of the projects and experimental protocols (a practice that is essential for carrying out confirmatory studies) (Wagenmakers et al., 2012; Nosek et al., 2018) and the embracement of open research practices (open data sharing) (Ferguson et al., 2014; Steckler et al., 2015; Gilmore et al., 2017) are also alternatives to improve reproducibility. Interestingly, it seems that just encouraging good research practices is not enough to assure compliance with the proposed guidelines (Baker et al., 2014; Hair et al., 2019). This suggests that the participation of research funding agencies is necessary as well as of peer reviewers and journal editors in demanding adherence to these directives (Kilkenny et al., 2009; Baker et al., 2014; Han et al., 2017; Hair et al., 2019).

In conclusion, paraphrasing Lazic et al. (2018), “There are few ways to conduct an experiment well, but many ways to conduct it poorly.” In our opinion, we, as a scientific community, have to be worried about the rigor of the experiments we are conducting and the quality of the studies we are producing. Publishing non-reproducible results (or reproducible noise) can lead to ethical, economic, and technological consequences leading to scientific discredit. Furthermore, poor reproducibility delays discovery and development and hinders the progress of scientific knowledge. Broad adherence and advanced training to principles of animal welfare and good experimental practices may elevate the standards of behavioral neuroscience. Finally, perhaps we, as the scientific community, should strive to refine our current animal models and focus our efforts in the development of new, more robust, ethologically relevant models that could potentially improve both the description of our reality and the translational potential of our basic research.

## Author Contributions

CML was responsible for the conceptualization of the opinion article. All authors were responsible for writing and revising the manuscript and read and approved the final manuscript.

## Conflict of Interest

The authors declare that the research was conducted in the absence of any commercial or financial relationships that could be construed as a potential conflict of interest.

## Publisher’s Note

All claims expressed in this article are solely those of the authors and do not necessarily represent those of their affiliated organizations, or those of the publisher, the editors and the reviewers. Any product that may be evaluated in this article, or claim that may be made by its manufacturer, is not guaranteed or endorsed by the publisher.

## Footnotes

1
https://www.nc3rs.org.uk

2
https://animalresearchtomorrow.org

## Key concepts (Glossary)

• Reproducibility: Obtaining the same results (similar effect sizes) as the original study by carrying out independent experiments (in different locations, laboratories, and research groups) in which the experimental procedures were as close as possible to the original study. Importantly, there is no need for the reproduction study to have exactly the same experimental design as the original study, for its result to be considered a reproduction. Also, as stated in Reproducibility Project and Cancer Biology, 2017, “if a replication reproduces some of the key experiments in the original study and sees effects that are similar to those seen in the original in other experiments, we need to conclude that it has substantially reproduced the original study.”
• Environmental enrichment: It consists in modifying the environment of animals by increasing perceptual, cognitive, physical, and social stimulation. In captive animals, it promotes improvements in the quality of life and animal welfare. Environmental enrichment represents an opportunity for the animals to evocate their ethological behaviors. For example, nocturnal animals usually escape bright environments by entering into shelters. In a future approach, it may represent a controlled naturalistic environment, such as a forest (as described in Landers et al., 2011).
• *Replace*: According to NC3Rs, it is “accelerating the development and use of models and tools, based on the latest science and technologies, to address important scientific questions without the use of animals.”
• *Reduce*: According to NC3Rs, it is “appropriately designing and analyzing animal experiments that are robust and reproducible and truly add to the knowledge base.”
• *Refine*: According to NC3Rs, it is “advancing animal welfare by exploiting the latest *in vivo* technologies and by improving understanding of the impact of welfare on scientific outcomes.”
• Physical models: According to Godfrey-Smith (2009), they are real systems purposely built to understand another real system.
• Animal models: According to Willner (1986), they are animal manipulations designed to model certain aspects (specific symptoms, for example) of a known disease.
• Behavioral tests: Paradigms designed to assess animal behavior. Commonly, they are used to evaluate the behavior of animals that were previously subjected to genetic, pharmacological, or environmental manipulations. In addition, they can also be used to investigation of the natural behavior of “naïve” animals.
• *Pseudoreplication*: It occurs when the researcher artificially inflates the number of experimental units by using samples that are heavily dependent on each other without correcting for it. Example 1) measuring multiple animals in a litter (after allocating all them to the same group) and treating them as independent samples (i.e., “*N*” equals the multiple measurements). Example 2) measuring two experimental animals that interacted with each other in a social interaction paradigm (i.e., the way that an animal behaves is influenced by the way the other one behaves, and vice-versa) and treating them as independent samples (i.e., “*N*” equals two).
• Experimental unit: It is the smallest entity that can be randomly and independently assigned to a treatment condition. For experimental units to be considered as genuine replications (i.e., the real “*N*”) they must not influence each other and must undergo experimental treatment independently. Its biological definition can change from one experiment to another (i.e., “*N* equals one” can be a single animal in an experiment and a pair of animals or even a whole litter in others).
• Exploratory studies: The ones that present more flexible experimental methods and designs. Their aim is not to reach statistical conclusions, but to gather information to the postulation of experimental hypotheses that must be tested and replicated through confirmatory studies before being assumed as strong evidence.
• Confirmatory studies: The ones that present clear predefined hypotheses to be tested and rigid methods to doing so (e.g., impartial assignment of experimental units to experimental groups, blinding during data collection and analysis, complete reporting of methods and results). Experimental design cannot be changed after the experiments are running. Must be presented in advance with well-defined biological effect sizes and statistical power, in addition to the *a priori* calculation of sample sizes. A clear example of confirmatory study is the Phase III of clinical trials in the process of vaccine development.
• Biological effect size: The calculated minimum effect size that is considered to be biologically relevant by the researcher.
• Confounding factors: Variables that can affect the outcomes that the researcher is measuring. Usually, they are not in the interest of the researcher and may assume categorical (e.g., litter, experimental blocks, and repeated measurements) or continual nature (e.g., age and body weight). Example 1) measuring siblings (after correctly allocating each one to a distinct experimental group) and analyzing their data as if they were not relatives. If the between-litter variation is higher than within-litter variation (i.e., the difference between families is higher than differences between siblings and, in this case, between experimental groups) the high data variability between litters could mask the effect of treatments. Example 2) Measuring drug-seeking behavior in a self-administration paradigm and analyzing the data without considering the basal motivation to self-administrating the drug (even when its variability was well controlled by randomization). If the basal motivation affects self-administration behavior the high within-group data variability (as a consequence of basal motivation variability) could mask the effect of treatments.
• Impossible predictions: Incorrectly estimating of values that are impossible to be observed for some types of data. It can occur when using linear models for analyzing count data (e.g., number of visible marbles, grooming, rearing, and pressures in a lever), where negative values are impossible to be observed but they can be often estimated by the analysis when the observed mean is low and/or the standard deviations are high.
• Directive 2010/63/EU: European Union Directive about animal welfare that established, among others, that “…all animals shall be provided with space of sufficient complexity to allow expression of a wide range of normal behavior. They shall be given a degree of control and choice over their environment to reduce stress-induced behavior.”
• Impoverished standard conditions: The conditions under which laboratory animals are bred by default in research facilities around the world. In general, the cages are too limited in space and contain only bedding (e.g., sawdust) plus water and food *ad libitum*. Improvements were made after some directives were established, but the “new standard” remains impoverished.
• Paradigm shift: According to Kuhn (1962), it is a fundamental change of concepts and experimental practices in science. Here, we adopted a more restricted use for this term. It represents a change in the experimental practices specifically for the environmental conditions of laboratory animals.
• Ethology: According to Merriam-Webster (https://www.merriam-webster.com/dictionary/ethology), it is the scientific study of animal behavior, usually with a focus on animal behavior under natural conditions. Viewing animal behavior as an evolutionarily adaptive trait.
• Ethological needs of the species: The basic natural needs (and also behavioral phenotypes) are distinct between each species. Based on the ethology concept, the environments where laboratory animals are kept or behaviorally tested must meet the intrinsic features of each species. Even though rats and mice are both rodents, they are different species and their characteristics and basic needs are not the same. This concept should be applied to all laboratory animals. For example, for ethical reasons, researchers do not submit rats to the tail suspension test. However, they do submit mice to the forced swim test (even though mice do not swim in nature).
